# Protective effect and localization by optical imaging of human renal CD133^+^ progenitor cells in an acute kidney injury model

**DOI:** 10.14814/phy2.12009

**Published:** 2014-05-11

**Authors:** Cristina Grange, Aldo Moggio, Marta Tapparo, Stefano Porta, Giovanni Camussi, Benedetta Bussolati

**Affiliations:** 1 Department of Medical Sciences, University of Torino, Torino, Italy; 2 Department of Molecular Biotechnology and Health Sciences, University of Torino, Torino, Italy

**Keywords:** AKI, biodistribution, mesenchymal stem cells, renal progenitor cells, stem cells

## Abstract

Recent approaches of regenerative medicine can offer a therapeutic option for patients undergoing acute kidney injury. In particular, mesenchymal stem cells were shown to ameliorate renal function and recovery after acute damage. We here evaluated the protective effect and localization of CD133^+^ renal progenitors from the human inner medulla in a model of glycerol‐induced acute tubular damage and we compared the results with those obtained with bone marrow‐derived mesenchymal stem cells. We found that CD133^+^ progenitor cells promoted the recovery of renal function, preventing tubular cell necrosis and stimulating resident cell proliferation and survival, similar to mesenchymal stem cells. In addition, by optical imaging analysis, CD133^+^ progenitor cells accumulated within the renal tissue, and a reduced entrapment in lung, spleen, and liver was observed. Mesenchymal stem cells were detectable at similar levels in the renal tissue, but a higher signal was present in extrarenal organs. Both cell types produced several cytokines/growth factors, suggesting that a combination of different mediators is involved in their biological action. These results indicate that human CD133^+^ progenitor cells are renotropic and able to improve renal regeneration in acute kidney injury.

## Abstract (precis)

In the present study, we found that administration of human CD133^+^ renal progenitors promoted renal repair after murine AKI, similar to mesenchymal stem cells. In addition, these cells showed a high renal localization evaluated by optical imaging analysis, and the production of renoprotective factors. Mesenchymal stem cells were detectable at similar levels in the renal tissue, but a higher signal was present in extrarenal organs.

## Introduction

The intrinsic ability of tissue repair and regeneration necessary to regain functionality after ischemic, toxic, or inflammatory insults is limited in the mammalian kidney. Acute kidney injury (AKI) remains a major cause of in‐hospital morbidity and mortality despite supply of supportive care, and possibly leads to chronic renal dysfunction (Goldstein et al. 2013). Recently, stem cell‐based therapy appeared as a new strategy to support renal repair, being bone marrow‐derived mesenchymal stem cells (MSCs) the most studied cell type and the most advanced in clinical development (Tögel et al. 2005). Injected bone marrow‐derived MSCs are known to display a renoprotective effect in models of acute kidney injury mainly through paracrine mechanisms (Bi et al. 2007).

In parallel, different experimental studies showed the potential application of adult renal progenitors derived both from mouse or human renal tissue for the treatment of AKI (Bussolati et al. 2005; Dekel et al. 2006; Sagrinati et al. 2006; Kinomura et al. 2008; Langworthy et al. 2009; Ronconi et al. 2009; Lee et al. 2010). In humans, CD133^+^ cells, characterized by features of nondifferentiated renal mesenchymal progenitors, were mainly identified in the Bowman capsule of glomeruli and in the proximal tubules (Bussolati et al. 2005; Sagrinati et al. 2006; Sallustio et al. 2010). These cortical CD133^+^ progenitor cells have been injected in murine models of glomerular toxicity and tubular necrosis (Bussolati et al. 2005; Sagrinati et al. 2006; Ronconi et al. 2009). In all experiments, injected CD133^+^ progenitors were detected into the renal injured tissue and were found to ameliorate renal function (Bussolati et al. 2005; Sagrinati et al. 2006; Ronconi et al. 2009). However, the localization of renal CD133^+^ cells in living animals and their biodistribution in the different organs has not yet been evaluated.

CD133^+^ progenitor cells were also detected in the inner medullary papilla region, being localized in the Henle's loop and in the S3 limb segments (Ward et al. 2011; Bussolati et al. 2012). CD133^+^ cells isolated from the renal medulla were shown to possess higher differentiative ability and stemness markers with respect to those in proximal tubules, possibly due to the maintenance of stem features in the hypoxic environment of the inner medulla (Bussolati et al. 2012). Therefore, it could be of interest to evaluate the in vivo effect and localization of the medullary CD133^+^ progenitor cells in models of AKI.

The present study was designed to investigate the effect of labelled CD133^+^ progenitor cells isolated from the human renal medulla after intravenous injection in mice with glycerol‐induced AKI. In addition, we report for the first time their biodistribution using optical imaging. The effect of renal progenitors was compared with that of bone marrow‐derived MSCs.

## Materials and Methods

### Cells and labeling procedure

Renal CD133^+^ progenitor cells were obtained from the normal portion of the papillary region of the inner medulla obtained from surgically removed kidneys (Bussolati et al. 2012), after approval of the Ethical Committee for the use of human tissue of the Department of Medical Sciences of the University of Torino. Briefly, tissue samples of approximately 3–5 mm^3^ were obtained at the papillary region of a renal pyramid. Tissue was rinsed with Hank's Balanced Salt Solution (Sigma, St. Louis, MO) and, after cutting, digested in 0.1% Collagenase type I (Sigma) for 45 min at 37°C. Tissue was subsequently forced through a graded series of meshes to separate the cell components from stroma and aggregates. The filtrate was pelleted by centrifugation. CD133^+^ cells were isolated by magnetic cell sorting, using the MACS system (Miltenyi Biotec, Bergisch Gladbach, Germany), and cells resuspended in expansion medium (EBM medium plus supplement kit; Lonza, Basel, Switzerland) without serum addition at a density of 1 × 10^5^ viable cells per cm^2^. Cell lines, obtained from different renal specimens were used between passage 2 and 5. Bone marrow‐derived MSCs were obtained from Lonza, cultured and characterized as previously described (Herrera et al. 2007; Bruno et al. 2009). Briefly, the MSCs were cultured in the presence of Mesenchymal Stem Cells Basal Medium (MSCBM, Lonza). All cell preparations at different passages of culture expressed the typical MSC markers: CD105, CD73, CD44, CD90, CD166, and CD146 and not hematopoietic markers like CD45, CD14, and CD34, evaluated by cytofluorimetric assay (Table 1). The adipogenic, osteogenic, and chondrogenic differentiation ability of MSCs was determined as previously described (Herrera et al. 2007).

**Table 1.** Phenotypic characteristics of CD133^+^ cells and MSCs.

Cell preparations	CD133	CD24	CD73	CD105	CD44	CD73	CD29	Vimentin	Pax2
CD133^+^ cells	97.5	84.9	95.5	6.4	96.2	71.3	81.9	>95	>95
MSCs	0	0	97.3	73.0	91.3	89.3	88.1	>95	0

The expression of the renal progenitor markers CD133 and Pax2 and of the mesenchymal markers CD73, CD105, CD44, CD29, and vimentin by the cell isolates used in the study was evaluated by cytofluorimetric analysis or immunofluorescence staining, as described (Bruno et al. 2009; Goldstein et al. 2013). Cells were tested between the second and the third culture passage. Data are expressed as % positive cells and are mean of four different cells lines for CD133^+^ cells and of three different cell lines for MSCs.

Cells were labeled with DiD (Ex: 640 nm; Em: 700 nm) (Molecular Probes, Life Technology, NY) solution without serum for 20 min at 37°C (Garrovo et al. 2013; Herrera et al. 2013). Cells were then washed in complete medium by centrifugation and cultivated for 24 h before injection in vivo. Cells were counted and viability tested by the trypan blue exclusion assay (Sigma).

### Optical imaging

All the studies were performed with IVIS 200 small animal imaging system (PerkinElmer, Waltham, MA) using excitation filter at 640 nm and emission filter at 700 nm. Identical illumination settings, such as exposure time (2 sec), binning factor (4), f/stop (2), and field of views (Herrera et al. 2013), were used for acquiring all images, and fluorescence emission was normalized to photons per second per centimeter squared per steradian (p/sec/cm^2^/sr). The color image represents the spatial distribution of fluorescence within the animal overlaid on black and white photographs of the mice, collected at the same time. Images were acquired whole body and on organs collected 48 h after cell injection. To control for the background photon emission, the obtained data were subjected to average background subtraction, using data captured with an excitation of 535 nm. Images were acquired and analyzed using Living Image 4.0 software (PerkinElmer) (Herrera et al. 2013). The fluorescence (p/sec/cm^2^/sr) was quantified in region of interest (ROI) draw freehand. Data were expressed as average radiance ± SD.

### Glycerol‐induced model of AKI

Animal studies were conducted in accordance with the National Institutes of Health Guide for the Care and Use of Laboratory Animals. The protocol was approved by the Committee of Bioethics of the University of Torino (Permit Number: 1.3.10). Mice were kept in our institutional animal facility under well‐controlled conditions of temperature, humidity with access to food and water ad libitum. AKI was induced in SCID mice (Charles River Laboratories, Lyon, France) by i.m. injection of 7.5 mL/kg glycerol (Sigma). Mice received 5 × 10^5^ CD133^+^ progenitors or MSCs in 150 *μ*L PBS or an equal volume of PBS as control (*n *=**6 mice/group for each experimental point) 24 h after the glycerol injection. Mice were sacrificed at different times after cell or vehicle administrations, and kidneys and samples for blood urea nitrogen (BUN) and creatinine determination were collected. The animals were monitored for activity and physical conditions every day. For biodistribution experiments, male CD1 nude mice were used (Charles River Laboratories). Mice were fed for 1 week with a special diet (AIN 79, Mucedola, Settimo Milanese, Italy) to reduce tissue autofluorescence. Twenty‐four hours after AKI damage, mice were treated with labeled CD133^+^ progenitors (*n *=**8) or MSCs (*n *=**6) or PBS (*n *=**8) and sacrificed 48 h after.

### Renal function

Blood samples for measurement of BUN and plasma creatinine were collected 0, 1, 3, 5, and 8 days after glycerol treatment. Serum creatinine was measured using a colorimetric microplate assay based on the Jaffe reaction (Quantichrom Creatinine Assay, BioAssay Systems, Hayward, CA). BUN was measured by direct quantification of serum urea with a colorimetric assay kit according to the instruction protocol (Quantichrom Urea Assay, BioAssay Systems).

### Morphological studies

For renal histology, 5 *μ*m‐thick paraffin kidney sections were routinely stained with hematoxylin and eosin. Luminal hyaline casts and cell lose (denudation of tubular basement membrane) were assessed in nonoverlapping fields (up to 10 for each section) using a 40× objective (high‐power field, HPF). Number of casts and tubular profiles showing necrosis were recorded in a single‐blind fashion (Bruno et al. 2009). Immunohistochemistry for the detection of proliferation of tubular cells was performed as previously described (Bruno et al. 2009). Briefly, kidney sections were subjected to antigen retrieval, and slides were blocked and labelled with 1:400 of monoclonal anti‐PCNA (Santa Cruz Biotechnology, Santa Cruz, CA). Immunoperoxidase staining was performed using 1:300 dilution of anti‐mouse HRP (Pierce, Rockford, IL). Scoring for PCNA‐positive cells was carried out by counting the number of positive nuclei per field in 10 randomly chosen sections of kidney cortex using 40× magnification.

Apoptosis was performed by TUNEL assay (Millipore, Vimodrone, MI) on slices of kidneys recovered after 48 h post cell injection (day 3), following the manufacturer's instruction. The number of apoptotic cells per field in 10 randomly chosen sections, using 200× magnification, was counted and expressed as mean ± SD.

Confocal microscopy analysis was performed on frozen sections for the detection of HLA‐class I, of Ki67 proliferation marker, and of pan cytokeratin. Sections were labelled with mouse anti‐HLA and rabbit polyclonal anti‐Ki67 antibodies (both from Abcam, Cambridge Science Park, UK) or with rabbit anti‐HLA (Santa Cruz) and mouse anti‐pan cytokeratin (Biolegend, San Diego, CA) or rat anti‐CD31 (Abcam). Alexa Fluor anti‐rabbit or anti‐mouse or anti‐rat antibodies (Molecular Probes, Leiden, the Netherlands) were used as secondary antibodies. Confocal microscopy analysis was performed using a Zeiss LSM 5 Pascal Model Confocal Microscope (Carl Zeiss International, Germany). Hoechst 33258 dye (Sigma) was added for nuclear staining. HLA‐positive cells were counted in 10 randomly chosen sections, using 400× magnification, and expressed as mean ± SD.

### Cytokine assay

To measure the cytokines produced by CD133^+^ cells and by MSCs, we performed a multiplex cytokine array based on fluorescently dyed microspheres (Bio‐Plex, Biorad, Hercules, CA). CD133^+^ cells and MSCs were cultured to a confluence of 90%. Cells were then cultured for 12 h in DMEM (Lonza) with 0.5% albumin (Sigma) and supernatant (conditioned medium) collected and frozen at −20°C after centrifugation (1200 rpm, 4°C, 5 min) to remove cellular debris. The cytokine assay was performed for three different isolated cells of CD133^+^ cells and MSCs.

### Statistical analysis

Results are generally expressed as mean ± SD. Statistical analysis was performed by ANOVA with Dunnett's multicomparison test. A *P‐*value of <0.05 was considered significant.

## Results

### Effect of CD133^+^ cells on the recovery of glycerol‐induced AKI and comparison with MSCs

**Figure 1.**
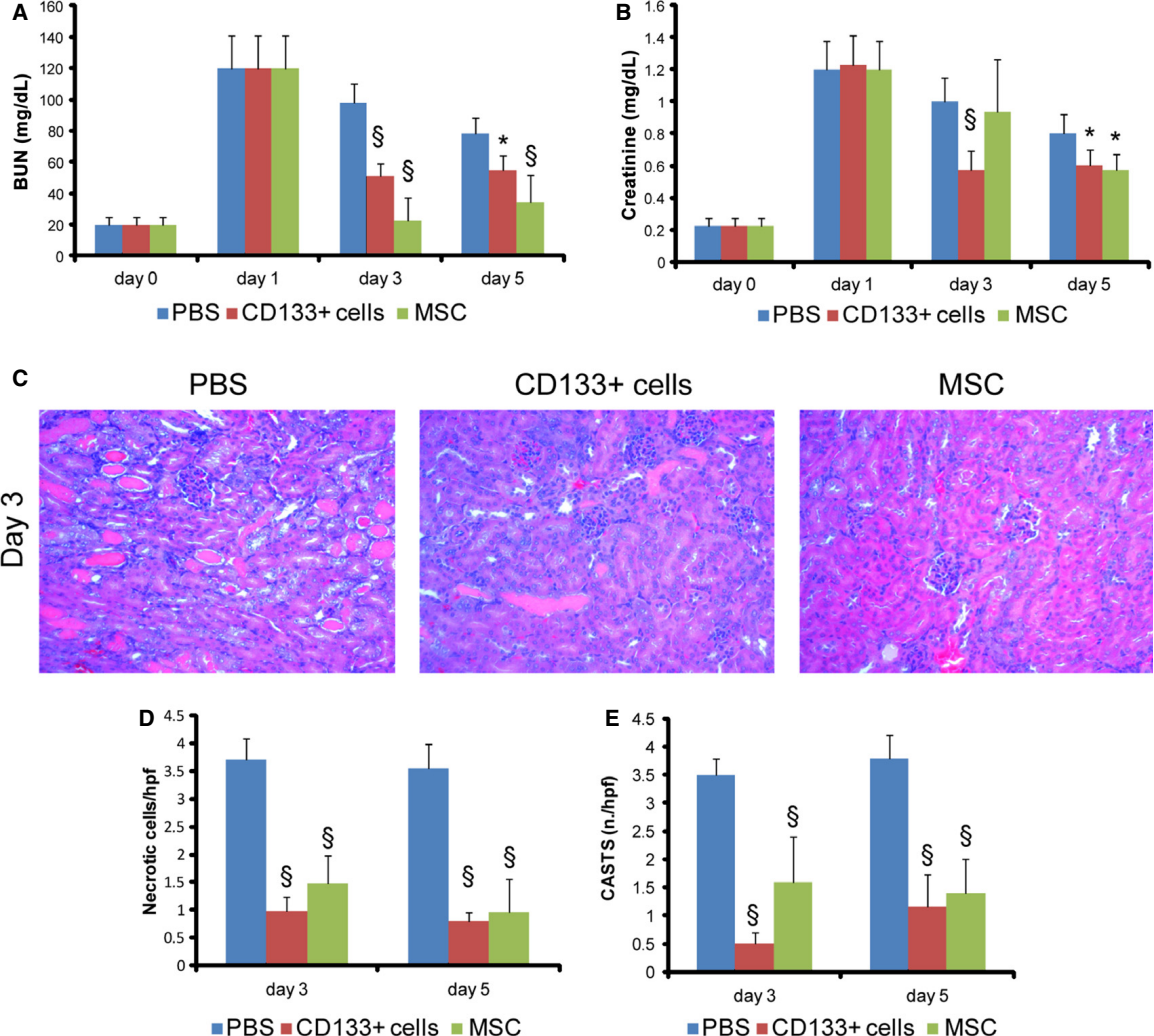
Effect of CD133^+^ cells or MSCs on renal function and morphology in AKI mice. Changes in renal function were measured by (A) BUN and (B) creatinine. Glycerol injection resulted in elevated BUN and creatinine levels with a peak at day 1. In animals injected with CD133^+^ cells at day 1 (arrow), BUN and creatinine levels showed a significant amelioration as soon as 48 h after injection (day 3) that was maintained at day 5. The effect of CD133^+^ cells was compared to that of MSCs injected at day 1. Data are mean ± SD of six SCID mice/group/experimental point. (C) Representative micrographs of renal tissue from AKI mice on day 3 after damage showing tubular necrosis, tubular protein casts and loss of brush border. Kidneys from mice treated with CD133^+^ cells or MSCs showed signs of recovery of tissue injury. Original magnification 200×. Count of tubular (D) necrosis and (E) tubular casts at day 3 and 5 after glycerol injection. Data are mean ± SD of the count of 10 fields/section in two sections/mouse. *n *=**6 mice for each experimental point were evaluated. ANOVA with Dunnett's multicomparison test was performed: *P <**0.05 stem cell treated versus PBS; ^§^*P *<**0.001 stem cell treated versus PBS.

CD133^+^ cells isolated from the human renal inner medulla were previously described to display progenitor phenotype and properties (Bussolati et al. 2012, 2013). CD133^+^ cells expressed several markers in common with MSCs (CD73, CD29, CD44, and vimentin, but not CD105) and, at variance of MSCs, the renal embryonic marker PAX2, indicating their renal origin (Table 1). To investigate the therapeutic effect of CD133^+^ cells derived from the human inner medulla, experimental AKI was induced in SCID mice by glycerol injection, as described before (Bussolati et al. 2005; Bruno et al. 2009). Intramuscular injection of glycerol causes rhabdomyolyses of the muscular tissue, thereby releasing large quantities of enzymes, myoglobin, and iron and causing renal tubular injury. After the glycerol injection, serum creatinine and BUN levels increased as soon as after 24 h, and remained elevated over the course of 5 days to normalize thereafter. One day after the induction of AKI, mice received 5 × 10^5^ CD133^+^ cells or MSCs intravenously via the tail vein. Control animals received only PBS. Mice injected with CD133^+^ cells exhibited a rapid amelioration of the renal function, as valuated by BUN and creatinine levels (Fig. 1A and B). In particular, a significant reduction in BUN and creatinine was observed at day 3 (48 h after treatment) and 5 with respect to that in animals treated with PBS alone. We next compared the effect of CD133^+^ cells with that of MSCs. As expected, MSCs rapidly protected against the development of AKI. The levels of BUN were lower in animals treated with MSCs with respect to CD133^+^ cells, whereas no difference was observed in creatinine levels (Fig. 1A and B).

### CD133^+^ cells and MSCs inhibited tubular necrosis and enhanced cell proliferation

**Figure 2.**
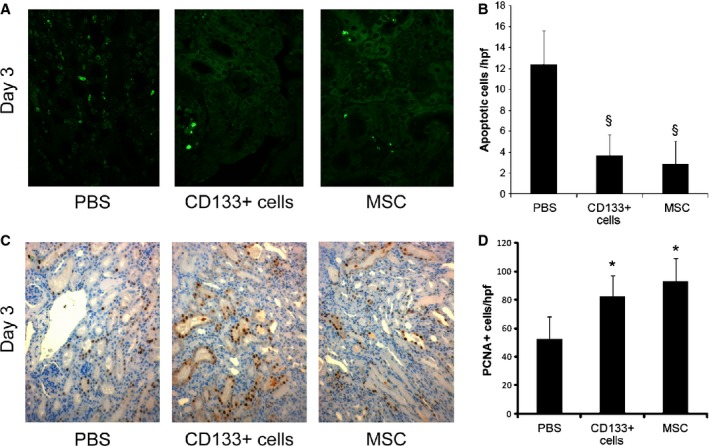
Tubular proliferation and apoptosis in AKI mice treated with CD133^+^ cells or MSCs. (A) Representative micrographs of TUNEL‐positive cells in AKI mice treated with PBS or stem cells. A significant reduction in apoptotic cells was observed in mice injected with CD133^+^ cells or MSCs. Original magnification 200×. (B) Graphs illustrating the quantification of TUNEL‐positive cells at day 3 in AKI mice treated with CD133^+^ cells or MSCs or injected with PBS. Data are expressed as mean ± SD of the count of 10 fields/section in two sections/mouse. *n *=**6 mice for each experimental point were evaluated. ANOVA with Dunnett's multicomparison test was performed: ^§^*P *<**0.001 stem cells versus PBS. (C) Representative micrographs showing PCNA‐positive cells in kidney tissue from AKI mice injected with PBS or with CD133^+^ cells or MSCs at day 3 after damage. Original magnification 200×. (D) Graphs illustrating the quantification of PCNA‐positive cells at day 3 in AKI mice treated with CD133^+^ cells or MSCs or injected with PBS as control. Data are expressed as mean ± SD of the count of 10 fields/section in two sections/mouse. *n *=**6 mice for each experimental point were evaluated. ANOVA with Dunnett's multicomparison test was performed: *P <**0.05 stem cells versus PBS.

On day 3 after injury, the histological evaluation of the renal tissue of glycerol‐injected mice showed diffuse epithelial damage characterized by necrosis and vacuolization of tubular epithelial cells. In addition, proximal tubular cells showed loss of brush border and intratubular protein casts (Fig. 1C). Animals treated with CD133^+^ cells or MSCs exhibited reduced tissue damage compared to PBS‐treated control animals as soon as at day 3 after glycerol injection (48 h after stem cell injection) (Fig. 1C). As assessed by morphometric evaluation of injury, stem cell‐injected animals had a significantly lower number of cast containing tubules and a lower number of necrotic tubules compared to PBS‐treated animals with AKI (Fig. 1D and E). Moreover, mice that received CD133^+^ or MSCs showed a significant reduction in apoptotic tubular cells compared with animals treated with PBS, as detected by TUNEL assay (Fig. 2A and B). No significant difference was observed between animals treated with CD133^+^ cells or MSCs.

We next evaluated the proliferation of proximal tubular cells in animals with AKI as a parameter of renal regenerative response. Treatment with CD133^+^ cells as well as with MSCs induced a significantly higher proliferation of tubular cells with respect to PBS‐treated controls at day 3 after injury (Fig. 2C and D).

### Intravenously‐injected CD133^+^ cells and MSCs localized in the kidney of AKI mice

**Figure 3.**
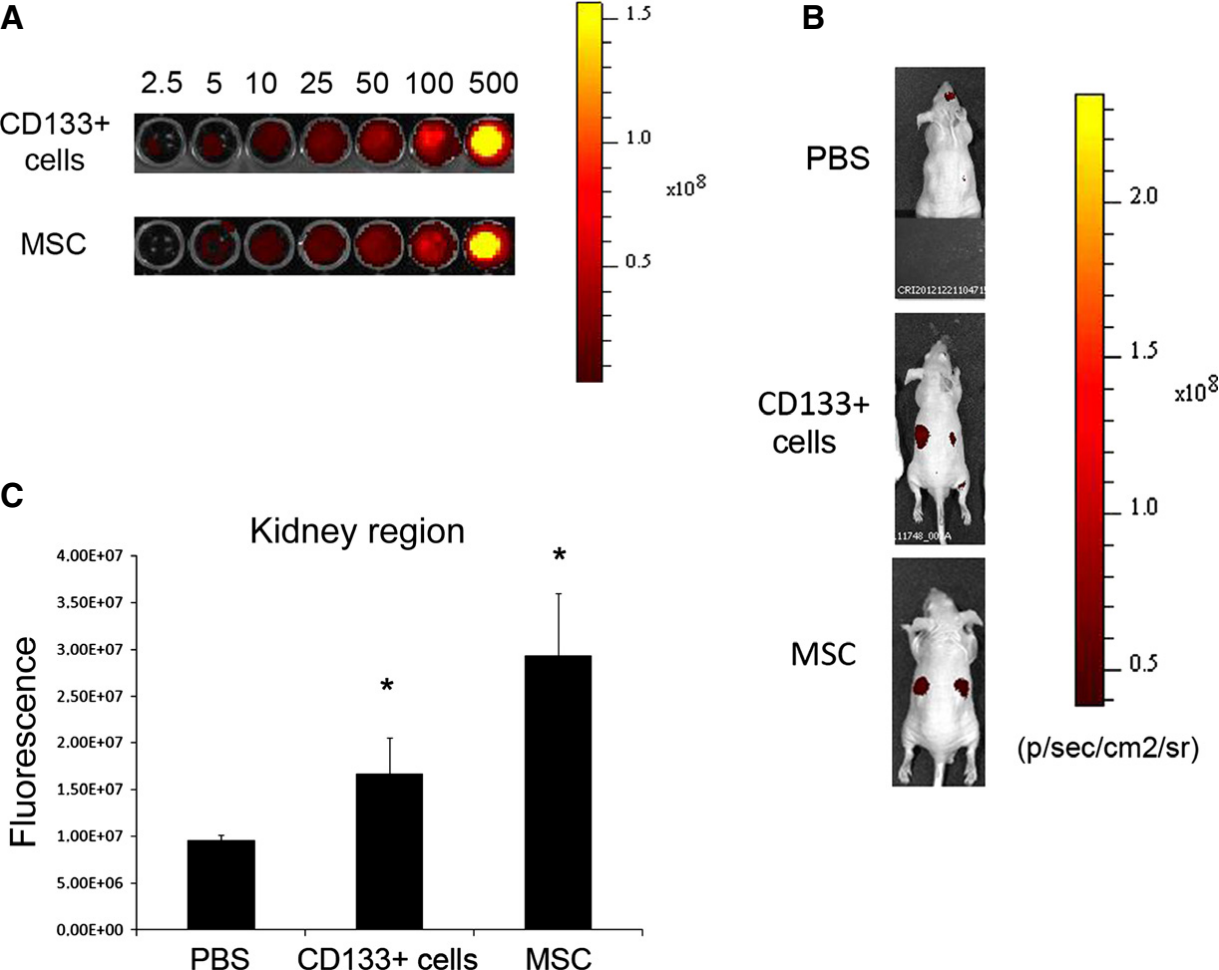
Localization of labeled stem cells in vivo by IVIS. (A) Representative fluorescence images of increasing number of DiD‐labeled stem cells (from 2.5 × 10^3^ to 500 × 10^3^) in an equal volume of 100 *μ*L PBS evaluated in vitro. (B) Representative fluorescence images of mice with AKI injected with DiD‐labeled stem cells or PBS evaluated at day 3 after damage. (C) Quantification of fluorescence intensity of the kidney region, calculated in ROI, expressed as the mean of average radiance ± SD of mice injected with labelled CD133^+^ cells or MSCs or PBS. Eight nude mice injected with PBS or CD133^+^ cells and six with MSCs were evaluated. ANOVA with Dunnett's multicomparison test was performed: *P <**0.05 stem cells versus PBS.

**Figure 4.**
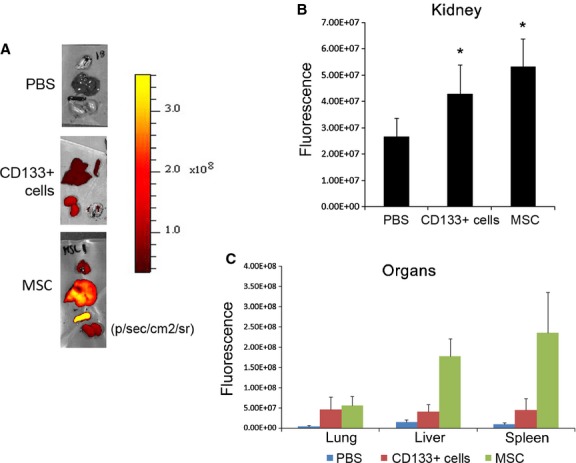
Ex vivo localization of labeled stem cells. (A) Representative fluorescence images of dissected organs of AKI mice sacrificed 48 h after injection of DiD‐labeled CD133^+^ cells or MSCs or PBS (day 3). (B) Quantification of the fluorescence intensity of kidneys, calculated in ROI, expressed as the mean of average radiance ± SD. Kidneys from eight nude mice injected with PBS or CD133^+^ cells and from six nude mice injected MSCs were evaluated. ANOVA with Dunnett's multicomparison test was performed: *P <**0.05 stem cells versus PBS. (C) Quantification of fluorescence intensity of organs (lung, liver, spleen), calculated in ROI, expressed as the mean of average radiance ± SD of organs from AKI mice injected with labelled CD133^+^ or MSCs or PBS.

The cell accumulation within the kidney was assessed by IVIS using CD133^+^ cells or MSCs labelled with DiD dye. A linear relation between cell number and fluorescence intensity was observed on labeled cells in vitro and a small number of cells, as low as 5 × 10^3^, was detectable. Equal number of CD133^+^ cells or MSCs generated comparable signals (Fig. 3A). Localization of the labeled cells was observed in whole body images and in ex vivo isolated organs at 48 h after the injection (day 3) (Figs. 3 and 4). As shown in Figure 3B and C, 48 h after intravenous injection CD133^+^ cells were detectable in the kidney with AKI damage. The fluorescence signal was higher than that of PBS‐treated animals and clearly detectable in the kidney region although lower than that obtained with labeled MSCs (Fig. 3B and C).

The analysis of isolated organs revealed a high localization of CD133^+^ cells within the kidney similar to that of MSCs (Fig. 4A and B). At variance, the localization within spleen and liver was significantly higher for MSCs than for CD133^+^ cells (Fig. 4C). Accumulation in lung was similar for the two cell types (Fig. 4C).

**Figure 5.**
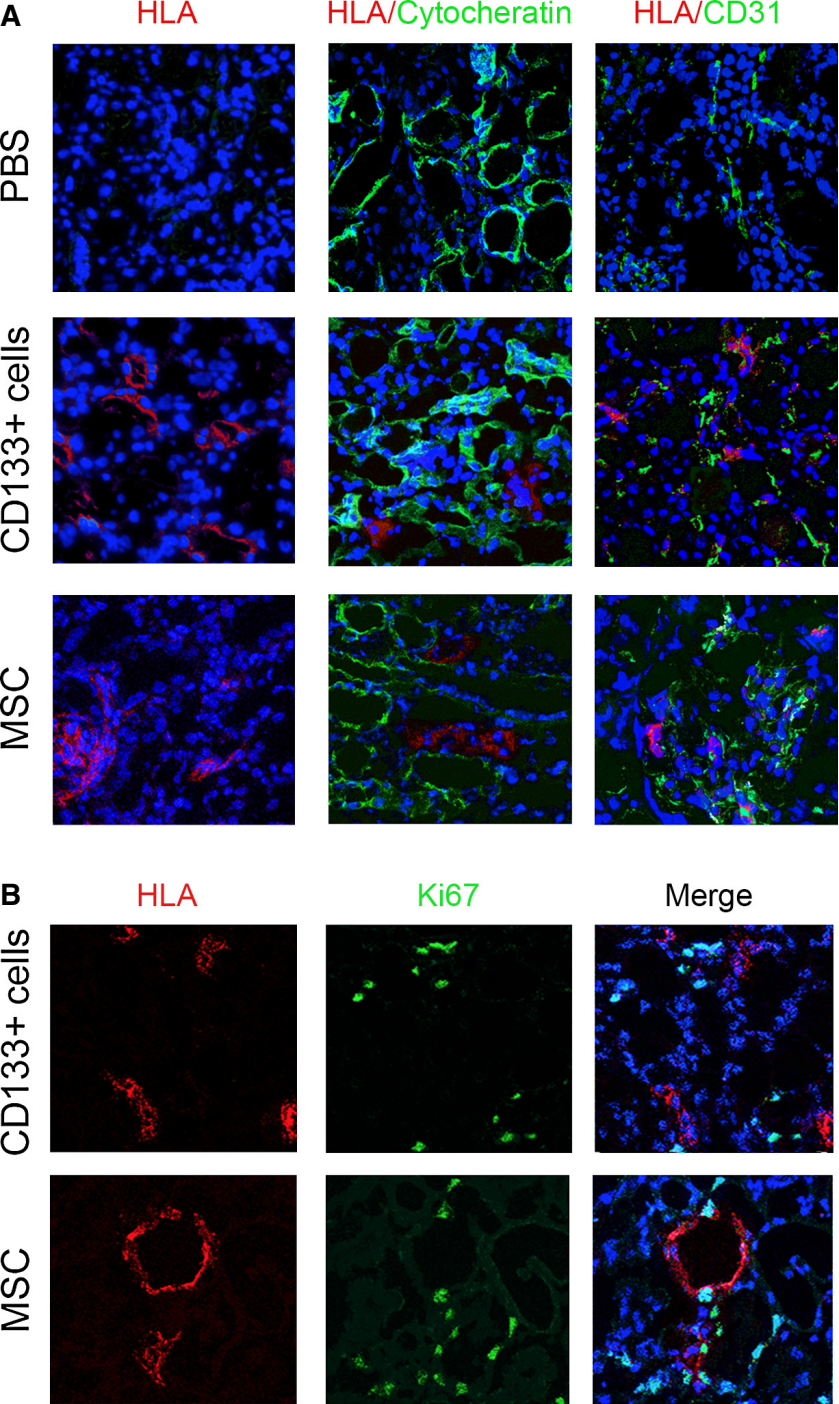
Detection of CD133^+^ cells or MSCs within the kidney of AKI mice. (A) Representative confocal micrographs showing the presence of CD133^+^ cells or MSCs within the kidney of mice with AKI at day 3 after damage as evaluated by HLA Class I (red). Localization of cytokeratin or CD31 positive cells is shown in green. (B) Representative confocal micrographs showing the presence of CD133^+^ cells or MSCs and of proliferating positive cells within the kidney of mice with AKI at day 3, as evaluated by HLA Class I (red) and Ki67 (green), respectively. Nuclei were counterstained with DAPI (blue). Original magnification 400×.

Immunofluorescence staining performed using human HLA Class I confirmed the engraftment of CD133^+^ cells and MSCs cells within the renal tissue after 48 h (Fig. 5). Cells were mainly present in renal interstitial tissue, but some CD133^+^ cells or MSCs were also detectable within glomeruli or tubular structures. No difference in cell number (12.9 ± 7.2 CD133^+^ cells; 13.4 ± 6.2 MSCs) or localization was detectable between the two stem cell sources. Colocalization studies with cytokeratin and CD31 showed absence of differentiation into epithelial or endothelial cells at this time point. In addition, double staining with Ki67 and HLA Class I showed that that the majority of human cells were not in a proliferative phase (Fig. 5B).

### Comparison of grow factors and cytokines produced by cultured CD133^+^ cells and MSCs

**Figure 6.**
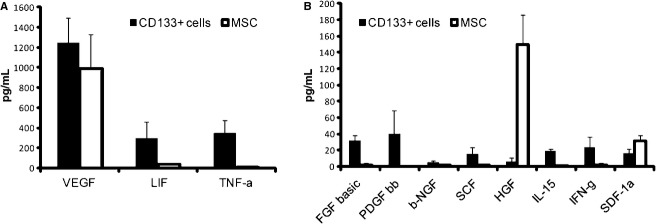
Cytokines/growth factors released in the conditional medium of CD133^+^ cells and MSCs. Evaluation of cytokines/growth factors released in the conditional medium of 1 × 10^6^ CD133^+^ cells and MSCs after 12 h incubation in RPMI plus 0.5% BSA. Cytokines were measured using a multiplex cytokine array. Data are the mean ± SD of three different cell lines. VEGF, vascular endothelial growth factor; LIF, leukemia inhibitory factor; TNF‐a, tumor necrosis factor α; FGF, fibroblast growth factor; PDGF, platelet‐derived growth factor; NGF, nerve growth factor; SCF, stem cell factor; HGF, hepatocyte growth factor; IFN‐g, interferon γ; SDF, stromal‐derived factor.

To investigate the possible mechanisms of repair induced by CD133^+^ cells and MSCs, we investigated the secretion of cytokines involved in tissue repair by a multiplex cytokine assay (Fig. 6). Both cell types released high levels of vascular endothelial growth factor. The main difference in cytokine/growth factors produced by CD133^+^ cells and MSCs was a preferential release of platelet‐derived growth factor, fibroblast growth factor, leukemia inhibitory factor, and tumor necrosis factor α by CD133^+^ cells with respect to MSCs. In addition, IL‐15, a kidney‐specific factors involved in renal differentiation (Giron‐Michel et al. 2013), was selectively released by CD133^+^ cells. At variance, MSCs showed a selective release of hepatocyte growth factor, known to be involved in epithelial cell regeneration (Matsumoto and Nakamura 2001).

## Discussion

In the present study, we investigated the potential of renal progenitor cell therapy in murine experimental AKI. We demonstrated that CD133^+^ progenitor cells isolated from the human renal inner medulla accelerate the recovery of AKI, similar to MSCs, prevented tubular cell necrosis and promoted cell proliferation. In addition, CD133^+^ progenitor cells were detectable by optical imaging within the renal tissue, with low entrapment in extrarenal organs.

Several experimental evidences indicate that administration of cells with stem/progenitor properties is effective in preventing renal damage and promoting its recovery after an ischemic or toxic insult. In this setting, MSCs from bone marrow or from fat and neonatal birth‐associated tissues (umbilical cord, placenta, and amniotic fluid) were proved to be successful (Aggarwal et al. 2013; Casiraghi et al. 2013).

A possible additional benefit in the exploitation of the regenerative properties of MSCs may derive from the use of tissue‐specific cells. Indeed, the gene expression profile comparative analysis of murine MSCs derived from kidney and bone marrow showed that renal MSCs express a selected pattern of genes possibly related to a memory of tissue origin (Pelekanos et al. 2012). This suggests that renal MSCs exhibit organ‐specific characteristics that could provide an advantage with respect to MSCs from unrelated organs. Cells with mesenchymal features and progenitor properties have been detected by mean of CD133 expression within different segments of the nephron (Bussolati et al. 2005, 2012; Sagrinati et al. 2006; Sallustio et al. 2010; Smeets et al. 2013). The physiological involvement of resident CD133^+^ progenitors in tissue regeneration after injury has been indicated by studies showing the increase and proliferation of these cells in renal transplanted kidneys undergoing ischemic damage and in biopsies of AKI patients (Loverre et al. 2008; Smeets et al. 2013). In addition, administration of CD133^+^ cells from tubules was reported to promote repair in AKI models, mainly by engraftment within murine tubules (Bussolati et al. 2005). Recently, a population of CD133^+^ cells has been detected in the renal medulla (Ward et al. 2011; Bussolati et al. 2012). We here show that CD133^+^ cells isolated from the human inner medulla and expanded in vitro promoted the recovery of renal function in a glycerol model of AKI. Moreover, CD133^+^ cells ameliorated the morphological features of the injured kidneys, reducing tubular necrosis and casts and promoting tubular cell proliferation and survival.

The effects observed after CD133^+^ cell infusion in terms of renal function were comparable to those of bone marrow‐derived MSCs, as reported previously (Herrera et al. 2004, 2007; Imberti et al. 2007; Morigi et al. 2008). At variance, we observed a clearly different biodistribution of the injected cells. Cells have been tracked using fluorescence methods with NIR dyes, such as the DiD dye, that provide efficient cell labeling without toxicity with a stable and strong signal. The use of small‐molecule fluorophores and, in particular, NIR molecules is a powerful tool to track stem cells for noninvasive visualization, giving the possibility to follow and to localize labeled cells in living whole‐body animal. These dyes were employed for in vivo cell, antibody, and extracellular vesicle detection (Zou et al. 2009; Boddington et al. 2011; Hood et al. 2011). Using optical imaging, previous experiments showed the possibility to localize and compare the distribution of MSCs in the kidney and other organs of AKI animals after intravenous or intra‐arterial injection (Tögel et al. 2008). We here confirmed using optical imaging that after injection in the tail vein, MSCs distributed in several organs, including kidney, lung, liver, and spleen. At variance CD133^+^ cells showed a high renal localization, but a reduced entrapment in lung, liver, and spleen.

It has been reported that paracrine factors released from MSCs localized in distant sites could be sufficient for their beneficial effects (Zhuo et al. 2013). In addition, as during AKI other organs such as the lung can be injured (Ahuja et al. 2012), the nonspecific homing of the injected cells might also contribute to systemic tissue repair. Nevertheless, the localization of the administered cells in the kidney might be more effective to obtain the desired therapeutic properties even when paracrine mechanisms are involved. In fact, within injured tissues, cells are exposed to an advantageous inflammatory environment that may influence their behavior leading to a bidirectional effect, from cells to the tissue and backward (Del Tatto et al. 2011).

Previous studies showed the integration of CD133^+^ cells isolated from cortex within murine renal structures as well as their proliferation (Bussolati et al. 2005; Angelotti et al. 2012). We here found that CD133^+^ cells, observed at an early time (48 h after their injection), were mainly localized in the interstitium and few cells were also detectable within renal tubular or glomerular structures. However, we only found a limited proliferation of CD133^+^ cells from the inner medulla, possibly indicating the involvement of paracrine mechanisms, as already described for MSCs (Imberti et al. 2007; Bruno et al. 2009; Gatti et al. 2011). This is also suggested by the lack of acquirement of epithelial or endothelial markers. Although further studies are required to analyze the possible integration of CD133^+^ cells from inner medulla in the murine kidney and their differentiation at later time, these results may suggest that these cells display differential properties with respect to those from cortex, as previously described (Bussolati et al. 2012). Indeed, CD133^+^ cells from the inner medulla were shown in vitro to be responsible for erythropoietin production (Bussolati et al. 2013). In the context of AKI, erythropoietin may play tissue‐specific roles unrelated to erythropoiesis such as modulation of angiogenesis and cell survival (Bahlmann and Fliser 2009; Wenger and Hoogewijs 2010). Moreover, several other factors released by CD133^+^ cells are considered of great importance in the modulation of renal regeneration. In particular, IL‐15 is a survival factor for tubular cells, involved in the maintenance of the renal epithelial phenotype (Giron‐Michel et al. 2013).

In conclusion, our results suggest that human isolated CD133^+^ renal progenitors could be suitable for cell therapy in acute tubular damage. In addition, these cells showed characteristics that might be related to their renal origin, such as their high renal localization and the production of renoprotective factors that could be exploited for renal regeneration. The possible clinical application of CD133^+^, both from cortex and medulla, might be limited by the availability of the organ source in comparison to multiple sources available for MSCs. However, the knowledge of the function of human CD133^+^ cells could lead to new pharmacological approaches targeting renal progenitors.

## Acknowledgments

We thank Claudia Cavallari for her technical support.

## Conflict of Interest

None declared.
